# Negative and Positive Psychosocial Factors in Relation to Cognitive Health in Older African Americans

**DOI:** 10.1093/geroni/igac019

**Published:** 2022-04-01

**Authors:** Maude Wagner, Anne-Josée Guimond, Laura D Kubzansky, Yingzhe Zhang, David A Bennett, Lisa L Barnes, Francine Grodstein

**Affiliations:** Rush Alzheimer’s Disease Center, Rush University Medical Center, Chicago, Illinois, USA; University of Bordeaux, Bordeaux, France; Department of Social and Behavioral Sciences, Harvard T.H. Chan School of Public Health, Boston, Massachusetts, USA; Lee Kum Sheung Center for Health and Happiness, Harvard T.H. Chan School of Public Health, Boston, Massachusetts, USA; Department of Social and Behavioral Sciences, Harvard T.H. Chan School of Public Health, Boston, Massachusetts, USA; Lee Kum Sheung Center for Health and Happiness, Harvard T.H. Chan School of Public Health, Boston, Massachusetts, USA; Department of Epidemiology, Harvard T.H. Chan School of Public Health, Boston, Massachusetts, USA; Harvard T.H. Chan School of Public Health, Boston, Massachusetts, USA; Rush Alzheimer’s Disease Center, Rush University Medical Center, Chicago, Illinois, USA; Department of Neurological Sciences, Rush Medical College, Chicago, Illinois, USA; Rush Alzheimer’s Disease Center, Rush University Medical Center, Chicago, Illinois, USA; Department of Neurological Sciences, Rush Medical College, Chicago, Illinois, USA; Rush Alzheimer’s Disease Center, Rush University Medical Center, Chicago, Illinois, USA; Department of Internal Medicine, Rush Medical College, Chicago, Illinois, USA

**Keywords:** African Americans, Cognitive function, Depressive symptoms, Linear mixed-effects models, Purpose in life

## Abstract

**Background and Objectives:**

Identifying potential intervention strategies to reduce cognitive decline, particularly among older African Americans at high risk for Alzheimer’s dementia, is critical. This study aimed to investigate whether depressive symptoms, purpose in life, and their interrelations are associated with cognitive decline in older African Americans.

**Research Design and Methods:**

We included older African Americans from the Minority Aging Research Study (*n* = 748) and Rush Memory and Aging Project (*n* = 109), without dementia at baseline. We assessed associations of depressive symptoms, purpose in life, and their interrelations, with baseline levels and change in global cognition using linear mixed-effects models.

**Results:**

At baseline, each unit increment in depressive symptoms was related to worse initial global cognition (mean difference = −0.03 standard unit; *p* = .003), while higher purpose in life was related to better cognition (mean difference = 0.12; *p* = .002). Further, participants with ≥1 depressive symptom who had a purpose in life score above the median appeared to have better global cognition (mean difference = 0.10; *p* = .01), compared to those with depressive symptoms but lower levels of purpose in life. However, we did not find relations of depressive symptoms or purpose in life with rates of cognitive decline over time, likely due to the modest follow-up.

**Discussion and Implications:**

In older African Americans, we found that lower depressive symptoms and greater purpose in life were independently related to higher initial levels of global cognition, but not cognitive decline. Preliminary findings of higher global cognition in individuals with depressive symptoms but greater purpose in life merit further investigation if purpose may eventually be considered as an intervention.

**Translational Significance:** This study examined how depressive symptoms, purpose in life, and their interrelations are related to cognitive health in a prospective study of older African Americans, who are severely understudied yet have high risk of cognitive impairment and dementia. Lower depressive symptoms and higher purpose in life at baseline were independently related to better baseline cognition. Further, in the presence of depressive symptoms, participants with higher scores (>median) of purpose in life had better global cognition. These preliminary findings could have important clinical and public health implications in terms of interventions to maintain cognitive function with aging in older African Americans.

## Background and Objectives

Older African Americans have high risk of Alzheimer’s dementia (AD) (Barnes & Bennett, 2014). Furthermore, over the next four decades, the proportion of African Americans among those aged 65 years and older is predicted to increase nearly 50% (Johnson & U.S. Census Bureau, 2020). Therefore, it is critical to identify potential intervention strategies to reduce cognitive decline, especially among African Americans.

A large body of research indicates that depression or significant depressive symptoms in later life are related to higher risk of developing dementia (Cherbuin et al., 2015). However, previous studies were conducted in cohorts of primarily White individuals (Berger et al., 1999; Dufouil et al., 1996; Geerlings et al., 2000; Köhler et al., 2010; Verdelho et al., 2013; Yaffe et al., 1999) and little is known regarding specific associations of depressive symptoms to cognitive aging in older African Americans (Turner et al., 2015; Wright et al., 2019). Depression generally presents differently in White than African American individuals (Bailey et al., 2019; Williams et al., 2007), among whom prevalence is lower but symptoms tend to be more chronic; thus, it is important to directly evaluate cognitive health in relation to depressive symptomatology within African American populations.

Additionally, there is increasing recognition that positive psychosocial assets, such as purpose in life, are not simply the absence of distress or other negative factors (Boehm & Kubzansky, 2012), but rather represent an independent aspect of psychosocial experiences. Indeed, although there is an inverse correlation between positive psychological assets and depressive symptoms, studies have suggested that correlations are small to moderate (Baselmans et al., 2018; Ryff & Keyes, 1995). Moreover, positive and negative psychological states have distinct biological underpinnings, further supporting the idea they reflect separate rather than mirrored constructs (Ryff et al., 2006). Thus, it is vital to expand research to focus on and actively evaluate relations of psychosocial assets to cognitive health. Purpose in life is a component of psychological well-being, and has been defined as the tendency to derive meaning from life experiences and to have goal orientation (Ryff & Keyes, 1995). While growing research (E. S. Kim et al., 2021) indicates that purpose in life may be related to less cognitive decline, and lower incidence of mild cognitive impairment (MCI) and AD, independent of depression, very few reports have examined associations in African Americans (G. Kim et al., 2019).

In research from our own group, we have previously reported that depressive symptoms are strongly related to cognitive decline and dementia in two primarily White cohorts (Bennett et al., 2018; Wilson et al., 2002, 2003). In an initial study, we found possible relations between certain aspects of depressive symptomatology (e.g., anhedonia, negative affect) with cognitive decline in the Minority Aging Research Study (MARS) of older African Americans (Turner et al., 2015). We have extensively documented that higher levels of purpose in life are associated with less cognitive decline and lower risk of dementia in primarily White cohorts (Bennett et al., 2018; Boyle et al., 2010, 2012), and most recently (Wilson et al., 2021), we found similar relations of purpose in life with MCI and dementia in both White and Black/mixed race participants in a cohort in Brazil. Here, we extend this research to: (a) examine the relation of depressive symptoms to cognition in African Americans, with a larger sample size than our previous MARS publication; and (b) examine the relation of purpose in life to cognition in African Americans. Further, to our knowledge, no previous research on cognition has explored interrelations of depressive symptomatology and purpose in life. Purpose in life has been considered in recent years as a target of intervention for preventing and treating late-life depression (Laird et al., 2019; Windsor et al., 2015); thus, we also examined interrelations of depressive symptoms and purpose in life to better understand their joint associations with cognition among 857 older African Americans.

## Research Design and Methods

### Study Populations

We utilized data from two ongoing, community-dwelling cohorts of aging and dementia. The MARS is a longitudinal cohort, which started in 2004 and enrolled African Americans aged 65 years or older without known dementia from churches, community-based organizations, and senior-subsidized housing facilities in the greater Chicago area (Barnes et al., 2012). To supplement the MARS sample, and to enlarge the sample size and enhance the statistical power of our analyses, we leveraged the Rush Memory and Aging Project (MAP), which started in 1997 and enrolled individuals (2% African American) without known dementia from metropolitan Chicago-area retirement and senior-subsidized housing facilities (Bennett et al., 2018). In both studies, recruitment, design, and operations are identical in essential details with a large common core of data at the item level. Follow-up exceeds 90% in both cohorts. All participants signed an informed consent form and both study protocols were approved by an Institutional Review Board of Rush University Medical Center.

### Eligible Participants

At the time of analyses, 774 older African Americans from MARS and 115 from MAP were enrolled. We excluded 24 participants who had dementia at baseline pursuant to clinical exam, 6 without complete neuropsychological testing at baseline, and 2 participants with missing data on purpose in life or depressive symptoms at baseline, leading to an analytic sample of 857 participants (748 MARS, 109 MAP).

### Global Cognitive Function Assessment

In both studies, each annual clinical evaluation included a uniform medical history, neurologic examination, and detailed neuropsychological performance testing (Barnes et al., 2012; Bennett et al., 2018). We used all cognitive data collected from study enrollment (i.e., beginning in 2004 for MARS and 1997 for MAP) to January 2021. A battery of 19 neuropsychological tests was administered annually and used to create a global composite measure of cognitive function in both cohorts. To create a global composite score, we converted raw scores on the 19 component tests across five cognitive systems to *z*-scores using the baseline mean and standard deviation (*SD*) from the combined parent studies.

In secondary analyses, we examined the five cognitive systems separately: episodic memory, semantic memory, working memory, perceptual speed, and visuospatial ability. Composite scores for each were calculated similarly as for the global composite. For all cognitive outcomes, higher scores indicated better cognitive function.

### Negative and Positive Psychosocial Factors at Baseline

#### Depressive symptoms

Depressive symptoms were assessed at baseline using a modified, 10-item version of the Center for Epidemiological Studies—Depression scale (CES-D). Participants were asked whether they experienced each of 10 symptoms much of the time in the past week (Wilson et al., 2002). The score is the total number of symptoms reported (range: 0–10).

#### Purpose in life

Purpose in life was assessed at baseline using a modified, 10-item measure derived from Ryff’s and Keyes’s Scales of Psychological Well-being. This measure conceptualizes purpose in life as an enduring attribute, and has high test–retest reliability (coefficient = 0.82; Ryff, 1989). Using a 5-point scale, participants rated their level of agreement with each item. Ratings for negatively worded items were reverse-scored, and items were averaged to yield a composite score (range: 1–5), with higher scores indicating greater purpose. The Cronbach’s coefficient α on the scale used in this study indicated a moderate level of internal consistency.

### Other Covariates at Baseline

Age, sex, years of education, and smoking status were obtained via self-report at baseline. Physical activity was self-reported using a modified version of the 1985 National Health Interview Survey; we considered the three overlapping items collected in both cohorts (i.e., walking, gardening, and exercising). We added the number of activities practiced during the previous 2 weeks. A composite measure of number of medical comorbidities was determined based on seven self-reported medical conditions: hypertension, diabetes, head trauma, heart disease, hypothyroidism, cancer, stroke. Finally, during their annual assessment, participants supplied all medications prescribed by a doctor and over-the-counter medications. These medications were identified and coded using the Medi-Span system (Medi-Span, Inc., Indianapolis, IN), and use of antidepressant medications was considered in some analyses.

### Statistical Analysis

We first considered continuous scores of depressive symptoms and purpose in life. We also created quartiles of purpose in life, and four categories of depressive symptoms (i.e., 0, 1, 2, 3+ symptoms) to consider possible threshold effects. Further, to better understand interrelations of purpose in life and depressive symptoms, we created a four-category variable: ≥1 depressive symptom/lower purpose; ≥1 depressive symptom/higher purpose; no depressive symptoms/lower purpose; no depressive symptoms/higher purpose. We chose a cut point of ≥1 depressive symptom because a small proportion of participants had depressive symptoms. In addition, because there are no standard cut points for lower and higher purpose in life, we dichotomized scores using the median of our analytic sample.

Using all repeated measures of global cognition collected over time, we used linear mixed-effects models which enabled estimation of the associations of baseline depressive symptoms and purpose in life with both initial level of global cognition and annual rate of cognitive decline. The linear mixed-effects model applies to longitudinal Gaussian outcomes, and accounts for the interindividual variabilities of repeated measures collected from the same individuals. Each model included a correlated individual random intercept and slope, with adjustment for age at baseline, sex, years of education, physical activity, number of comorbidities (on both the intercept and the slope), and an indicator for cohort; we also controlled for depressive symptoms when examining purpose in life, and purpose in life when examining depressive symptoms. Finally, we considered that religious service attendance could be related to depressive symptoms or to purpose in life, and potentially be a confounder in the relation of each of these to cognition. Participants in both cohorts had provided information on their frequency of religious service attendance; however, because the data showed no difference in the distribution of religious service attendance by depressive symptoms or purpose in life, we did not further examine religious service attendance here.

### Secondary Analyses

In secondary analyses, as described above, we explored associations of purpose in life and depressive symptoms with each of the five cognitive systems. Second, because there are some differences between African Americans from MARS and MAP (e.g., MARS participants had more mean years of education; Supplementary Table S1), we separately examined the 748 participants in MARS. Third, to help address any potential residual confounding in analyses of purpose in life, we conducted analyses of purpose in life among participants with no depressive symptoms at baseline. Finally, although few participants (*n* = 58) used antidepressant medication at baseline, we also created a model adding a covariate for use of antidepressant medications (on both the intercept and slope).

Model validation was performed graphically and analytically, and there was no evidence of multicollinearity (i.e., variance inflation factors < 2). Analyses were conducted using R software version 4.0.3 (R Foundation for Statistical Computing, Vienna, Austria) and we used the lme function of nlme R package version 3.1-149 for linear mixed-effects models.

## Results

The majority of participants were women (78%), mean age was 73 (*SD* = 6) years, and participants had a mean of 15 (*SD* = 3) years of education (data not shown in table). At baseline, approximately 42% of individuals had no depressive symptoms, with 20% reporting ≥3 symptoms, and mean score for purpose in life was 4 (*SD* = 0.5). Depressive symptoms were only modestly and negatively correlated with purpose in life (Pearson correlation *r* = −0.34). During a mean follow-up of 6 years (*SD* = 5, range = [1; 17]), the mean number of cognitive assessments was 6 (*SD* = 4, range = [1; 17]).

When we considered characteristics of participants according to baseline levels of depressive symptoms (i.e., ≥1 symptom vs none), or purpose in life (i.e., above vs below the median; Table 1), those with ≥1 depressive symptom had slightly lower levels of purpose in life than those with no symptom (mean scores on purpose = 3.8 [*SD* = 0.5] vs 4.0 [*SD* = 0.4], respectively). Additionally, those with depressive symptoms were more likely to be women, had somewhat fewer years of education, and higher prevalence of antidepressant medications, although few participants used these medications. Participants with depressive symptoms had also generally worse levels of cognitive function at baseline than those without symptoms. When comparing participants with higher and lower purpose in life at baseline, those with higher purpose had fewer depressive symptoms than those with lower purpose (mean depressive symptoms = 0.9 [*SD* = 1.4] vs 1.8 [*SD* = 2.0], respectively). Further, those with higher levels of purpose in life were younger, had more years of education, reported less use of antidepressant medication, and had better performance in global cognition and each cognitive system (Table 1).

**Table 1.** Baseline Characteristics of Participants, According to Levels of Depressive Symptoms and Purpose in Life

Characteristics	Depressive symptoms		Purpose in life	
	≥1 (*n* = 501)	None (*n* = 356)	Higher (above median) [3.9–5] (*n* = 440)	Lower (below median) [2–3.8] (*n* = 417)
Mean score of purpose in life (*SD*)	3.8 (0.5)	4.0 (0.4)	4.2 (0.3)	3.5 (0.3)
Mean number of depressive symptoms (*SD*)	2.3 (1.7)	0	0.9 (1.4)	1.8 (2.0)
Mean age (*SD*), years	73.2 (6.6)	73.6 (6.2)	72.4 (5.8)	74.4 (6.9)
Female, %	80	74	77	78
Mean education (*SD*), years	14.4 (3.4)	15.0 (3.5)	15.6 (3.5)	13.7 (3.1)
Antidepressant medication, %	9	4	5	9
Mean number of comorbidities (*SD*)	1.6 (1.0)	1.5 (1.0)	1.5 (1.0)	1.7 (1.0)
Mean number of reported physical activities^a^ (*SD*)	1.1 (0.9)	1.2 (0.9)	1.2 (0.9)	1.0 (0.9)
Smoking status, %				
Never	48	50	51	47
Former	43	44	42	45
Current	9	6	7	9
Mean baseline cognitive function (*SD*), *z*-scores				
Global cognition	−0.13 (0.54)	−0.01 (0.50)	0.06 (0.49)	−0.22 (0.53)
Episodic memory	0.01 (0.59)	0.10 (0.59)	0.17 (0.55)	−0.08 (0.60)
Semantic memory	−0.17 (0.80)	−0.01 (0.70)	0.03 (0.70)	−0.24 (0.79)
Visuospatial ability	−0.39 (0.82)	−0.30 (0.79)	−0.21 (0.75)	−0.51 (0.83)
Perceptual speed	−0.15 (0.77)	−0.02 (0.74)	0.08 (0.74)	−0.28 (0.74)
Working memory	−0.22 (0.77)	−0.05 (0.72)	−0.04 (0.73)	−0.27 (0.76)

*Notes*: *SD* = standard deviation.

^a^Number of physical activities (among walking, gardening, and exercise) practiced within the past 2 weeks.

### Depressive Symptoms

**Figure 1.**
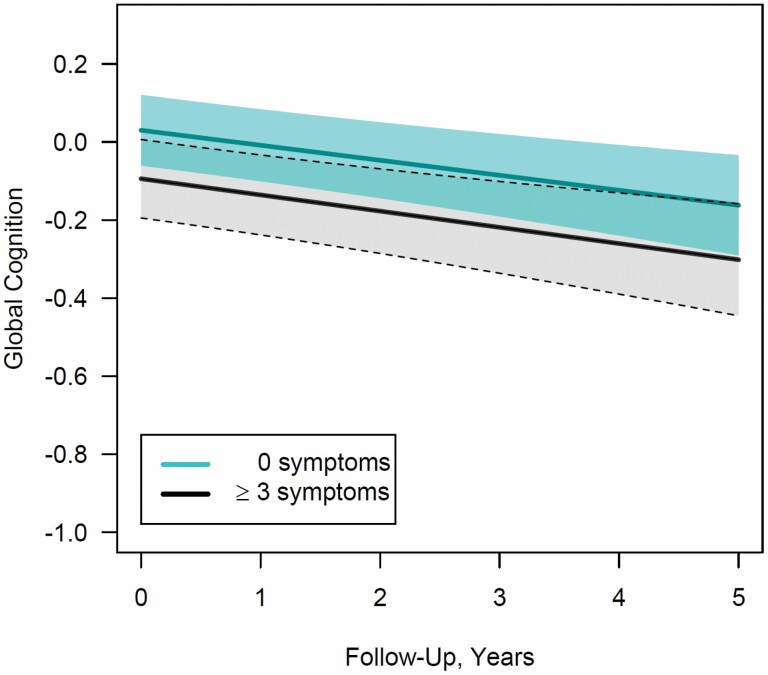
Depressive symptoms at baseline and global cognitive function over time. *Notes*: For no depressive symptoms and three or more symptoms, trajectories of global cognitive function were plotted for the most common profile of covariates in the study sample (i.e., female in Minority Aging Research Study, 73 years of age, 15 years of education, two medical comorbidities, purpose in life score of 4). Shading represents the 95% pointwise confidence intervals.

When considering number of depressive symptoms as a continuous variable, we found that each increment in the number of symptoms was associated with worse mean initial level of global cognition, after multivariable adjustment (mean difference = −0.03 standard units, standard error [*SE*] = 0.01; *p* = .003; Table 2). When comparing participants with none versus 1, 2, or ≥3 depressive symptoms, we found no relation with 1 symptom (mean difference = −0.05, *SE* = 0.04; *p* = .2), while those with 2 or with ≥3 symptoms had significantly worse initial levels of cognition (2 symptoms: mean difference = −0.10, *SE* = 0.05; *p* = .046; ≥3 symptoms: mean difference = −0.12, *SE* = 0.04; *p* = .006). During follow-up, the level of cognitive function was consistently worse for those with more depressive symptoms (Figure 1); however, the annual rate of change, or slope of cognitive decline, was similar regardless of the number of depressive symptoms (all *p* > .6; Table 2), possibly due to the modest average follow-up. That is, it often requires long periods of follow-up to detect meaningful changes in cognitive trajectories, which can remain relatively flat over modest periods of time.

**Table 2.** Multivariable-Adjusted Mean Differences in Baseline Global Cognition and Global Cognitive Decline, According to Levels of Depressive Symptoms and Purpose in Life at Baseline

Model term	*n* (%)	Initial level		Rate of decline	
		Mean difference (*SE*)	*p*	Mean difference (*SE*)	*p*
Depressive symptoms^a^					
Continuous	857	−0.03 (0.01)	.003	−0.001 (0.002)	.6
0 symptoms	356 (41.5)	Ref.	—	Ref.	—
1	237 (27.7)	−0.05 (0.04)	.2	−0.0002 (0.01)	.9
2	91 (10.6)	−0.10 (0.05)	.046	−0.0001 (0.01)	.9
≥3	173 (20.2)	−0.12 (0.04)	.006	−0.004 (0.01)	.7
Purpose in life^b^					
Continuous	857	0.12 (0.04)	.002	0.001 (0.01)	.9
Quartile 1 (2.0–3.6)	241 (28.1)	Ref.	—	Ref.	—
Quartile 2 (3.7–3.9)	254 (29.6)	0.05 (0.04)	.3	−0.0001 (0.01)	.9
Quartile 3 (4.0–4.1)	180 (21.0)	0.11 (0.04)	.01	−0.003 (0.01)	.7
Quartile 4 (4.2–5.0)	182 (21.2)	0.14 (0.05)	.002	0.004 (0.01)	.7

*Notes*: *SE* = standard error.

^a^Adjusted for sex, age at baseline (continuous, in years), education (continuous, in years), cohort, the number of medical comorbidities, physical activity, and purpose in life at baseline.

^b^Adjusted for sex, age at baseline (continuous, in years), education (continuous, in years), cohort, the number of medical comorbidities, physical activity, and the number depressive symptoms at baseline.

### Purpose in Life

**Figure 2.**
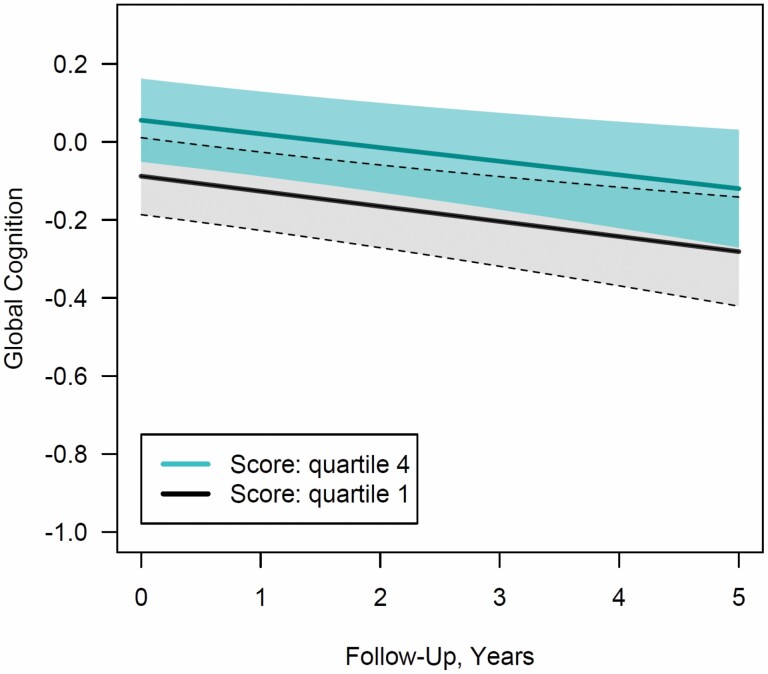
Purpose in life at baseline and global cognitive function over time. *Notes*: For the highest and lowest quartiles of purpose in life, trajectories of global cognitive function were plotted for the most common profile of covariates in the study sample (i.e., female in Minority Aging Research Study, 73 years of age, 15 years of education, two medical comorbidities, one depressive symptom reported on the Center for Epidemiological Studies—Depression scale). Shading represents the 95% pointwise confidence intervals.

When we examined purpose in life as a continuous score, each one-unit increment in purpose in life was strongly associated with higher mean initial levels of global cognition, after multivariable adjustment (mean difference = 0.12, *SE* = 0.04; *p* = .002; Table 2). When considering quartiles of purpose in life, we found no relation for the second quartile of purpose to cognitive function (mean difference = 0.05, *SE* = 0.04; *p* = .3) compared to the bottom quartile, whereas the third and fourth quartiles were both associated with higher baseline cognitive function (third quartile: mean difference = 0.11, *SE* = 0.04; *p* = .01; fourth quartile: mean difference = 0.14, *SE* = 0.05; *p* = .002). As demonstrated in Figure 2, those with higher levels of purpose in life at baseline appeared to have higher levels of global cognition at repeated time points during follow-up; however, purpose in life was not associated with slopes of cognitive decline (all *p* > .7; Table 2).

### Interrelations of Depressive Symptoms and Purpose in Life

To better understand how negative and positive psychosocial factors may interrelate, we compared participants across combined categories of depressive symptoms and purpose in life (Table 3). Compared to those with ≥1 depressive symptom and lower purpose in life at baseline, those with ≥1 depressive symptom and higher purpose had significantly better baseline global cognition (mean difference = 0.10, *SE* = 0.04; *p* = .01). When we considered the “opposite” combination—those with no depressive symptoms and lower purpose—we found a borderline significant association with better cognition (mean difference = 0.08, *SE* = 0.05; *p* = .09). Finally, as expected, there were particularly strong relations with global cognition for those with no depressive symptoms/higher purpose in life (mean difference = 0.19, *SE* = 0.04; *p* < .0001), compared to ≥1 depressive symptom/lower purpose. We found no associations of the combined categories of purpose and depressive symptoms at baseline with cognitive decline over time (all *p >* .3; Table 3; Supplementary Figure S1).

**Table 3.** Multivariable-Adjusted Mean Differences in Baseline Level of Global Cognition and Global Cognitive Decline, According to Cross-Categories of Depressive Symptoms and Purpose in Life at Baseline

Model term	*n* (%)	Initial level		Rate of decline	
		Mean difference (*SE*)	*p*	Mean difference (*SE*)	*p*
Categories of purpose in life and depressive symptoms^a^	857		<.0001		.7
≥1 depressive symptom, lower purpose	284 (33.1)	Ref.	—	Ref.	—
≥1 depressive symptom, higher purpose	217 (25.3)	0.10 (0.04)	.01	−0.01 (0.01)	.4
No depressive symptoms, lower purpose	133 (15.5)	0.08 (0.05)	.09	−0.01 (0.01)	.3
No depressive symptoms, higher purpose	223 (26.0)	0.19 (0.04)	<.0001	0.004 (0.01)	.6

*Notes*: *SE* = standard error.

^a^Adjusted for sex, age at baseline (continuous, in years), education (continuous, in years), cohort, physical activity, and the number of medical comorbidities at baseline.

### Secondary Analyses

When examining associations between depressive symptoms and each cognitive system separately, we found significant relations with baseline levels of episodic memory, semantic memory, and perceptual speed (Supplementary Table S2). For purpose in life, we generally found associations across most cognitive systems (Supplementary Table S3), suggesting that purpose in life may have quite broad relations with cognitive health. When examining the MARS cohort only, all findings remained similar (Supplementary Table S4) to those reported above. In analyses excluding those with any depressive symptoms at baseline in the combined cohorts of MARS and MAP, we still found strong associations of purpose in life with cognitive function (continuous score of purpose: mean difference = 0.23, *SE* = 0.06; *p* < .0001; Supplementary Table S5). Finally, addition of antidepressant medications to multivariable models for depressive symptoms did not change findings (results not shown).

## Discussion and Implications

In this study of >800 older African Americans, depressive symptoms and purpose in life at baseline were both independently associated with the initial level of global cognition. Further, both depressive symptoms and purpose in life were related to most cognitive systems we examined, indicating robust associations with cognitive function. Additionally, in the presence of depressive symptoms, individuals with higher purpose in life (>median) had better cognition than those with depressive symptoms and lower purpose in life, an interesting preliminary finding that may support future evaluation of psychological therapies for depression in older persons that incorporate enhancement of well-being, as a way to help maintain cognitive health in aging. However, neither depressive symptoms nor purpose in life were related to annual rate of cognitive decline over a mean follow-up of 6 years.

Depressive symptomatology has been extensively analyzed in relation to cognitive function in later life, although we are aware of only two prior studies considering older African American adults, from the Healthy Aging in Neighbourhoods of Diversity across the Life Span (HANDLS) study (Wright et al., 2019) and our own previous research in the MARS cohort (Turner et al., 2015). Our results here are consistent with these earlier reports, in finding that older African Americans with depressive symptoms at baseline had poorer initial levels of global cognition, episodic memory, semantic memory, and perceptual speed (Turner et al., 2015; Wright et al., 2019). In addition, our finding that depressive symptoms were not associated with accelerated decline in either global cognition or cognitive systems is similar to the earlier work in MARS, which had a smaller sample (*n* = 298) followed for an average of 5 years (Turner et al., 2015). Nevertheless, one large study with 5.3 mean years of follow-up, and 62% African Americans (*n* > 2,700 African Americans; Wilson et al., 2004), found that higher CES-D in later life was related to cognitive decline over time; the authors reported no significant interaction of race with depressive symptoms, indicating results were similar across racial groups. It is plausible that with additional follow-up and continued recruitment of new participants, we will also observe a relation of depressive symptoms to cognitive decline in MARS and MAP.

In terms of mechanisms, depressive symptoms in older adults are associated with hippocampal–pituitary–adrenal axis dysregulation and subsequent increased cortisol production (Byers & Yaffe, 2011). Research on the hippocampal–cortisol pathway comes from animal studies, which indicate that chronic stress can lead to hippocampal atrophy (Cereseto et al., 2006) and memory impairment (Park et al., 2008). Further, associations of depressive symptoms with smaller hippocampal volume in humans have been reported (Elbejjani et al., 2015), including a study with nearly 50% African Americans (Brown et al., 2014). Depression has also been related to higher levels of biomarkers of oxidative stress, inflammatory markers (Weisenbach et al., 2019), and insulin resistance (Diniz et al., 2018), all pathways implicated in dementia.

Consistent with previous studies (cross-sectional [Lewis et al., 2017; Zahodne et al., 2018] and longitudinal [Boyle et al., 2010; G. Kim et al., 2019]) on primarily White older participants, we found that older African Americans with greater purpose in life had better initial level of global cognition. However, we were not able to detect associations between purpose in life and cognitive decline. In a larger study, G. Kim and colleagues (2019) examined the interaction between race and time during a 6-year period and found greater purpose was related to less cognitive decline, including nearly 1,700 older Black adults. Thus, again, it is likely that purpose in life is related to cognitive decline in African Americans as well when studies have larger sample and/or longer follow-up.

There are several pathways by which purpose in life may be related to cognitive health. First, older individuals with greater purpose in life appear more likely to have better health behaviors; previous research has suggested that purpose in life is related to more use of preventive health services (E. S. Kim et al., 2014), to healthy dietary habits and regular exercise (Hirooka et al., 2021; E. S. Kim et al., 2020), which have been associated with cognition and dementia risk (Kivipelto et al., 2018). Another possible pathway is that purpose is related to biological processes relevant to cognitive function (Ryff et al., 2006). For example, greater purpose has been linked to lower interleukin-6 plasma concentration in older women (Friedman et al., 2007), and lower levels of hemoglobin A1c (Boylan et al., 2017; Hafez et al., 2018); inflammation and glucose regulation may be related to cognitive health (Darweesh et al., 2018; Yaffe et al., 2003). Further, purpose in life has been related to lower risk of clinical cardiovascular disease (E. S. Kim, Sun, Park, Kubzansky, et al., 2013) and stroke (E. S. Kim, Sun, Park, & Peterson, 2013), as well as less cerebrovascular pathology (Yu et al., 2015). Thus, it is possible that purpose in life is associated with better cognitive functioning through these biologic pathways.

Finally, our work here extends previous research by considering interrelations of depressive symptoms and purpose in life. In particular, we found apparently better cognitive function in those with depressive symptoms when individuals also had higher levels of purpose in life. Our results highlight the broad value of understanding how negative and positive psychological factors interrelate. Further, in terms of interpretation, our initial finding that those with both higher purpose in life as well as depressive symptoms had apparently better cognition than those with depressive symptoms and lower purpose in life may have future clinical implications. In particular, our results point to the potential importance to cognitive health of trying to evaluate psychological interventions for depression that aim to enhance purpose in life. For example, acceptance and commitment therapy has been found efficacious in reducing depression, and aims to help individuals live meaningful lives by increasing their engagement in activities that are consistent with their values (Bai et al., 2020). Cognitive-behavioral therapy and meaning-centered psychotherapy are other interventions that can be used to simultaneously target purpose in life and depressive symptoms (Holland et al., 2015). Future research examining such interventions in relation to cognitive function in older adults with depressive symptoms could be important.

However, limitations of our study warrant consideration. First, our primary findings were cross-sectional, and thus we cannot determine whether fewer depressive symptoms or higher purpose in life led to better cognition, or whether better cognition might result in fewer depressive symptoms and higher purpose. As we noted earlier, we will continue to follow participants and to recruit additional participants to further assess prospective relations. At least for depression, substantial previous research has found that depressive symptoms many years in the past are related to subsequent dementia risk (Byers & Yaffe, 2011), indicating that depressive symptoms are likely a risk factor rather than a prodromal symptom. Further, in our other studies (Wilson et al., 2003), depressive symptoms were not related to AD neuropathology, suggesting that depression is not simply an early manifestation of the pathologies causing dementia.

Regarding other limitations, although we controlled analyses for demographics and baseline medical conditions, residual confounding is possible in observational studies. Additionally, a possible limitation is that we combined two cohorts to maximize our sample size and statistical power. However, both cohorts had nearly identical recruitment and data collection procedures, and we confirmed that the overall findings in the combined cohorts were qualitatively consistent with findings in MARS alone (MARS represents the larger set of African American participants). Further, for the analyses of depressive symptoms, over 40% of participants had no symptoms, and the minority (20%) had three or more symptoms. Thus, we were not able to examine how severe symptoms were associated with cognitive health; however, the strong association we found of even low levels of depressive symptoms with cognition indicates the potential importance of depressive symptomatology to cognition, and the need to carefully assess and monitor symptoms in African Americans as a potential approach to maintain cognitive health. Finally, with the low prevalence of significant depressive symptoms in these cohorts, our analyses of interrelations of depressive symptoms with purpose in life may not be directly applicable to those with severe depression.

There are major strengths of this study, including the groups of well-characterized older African Americans, with annual, comprehensive neuropsychological testing, validated scales of purpose in life and depressive symptoms, and a high rate of follow-up. Finally, by exploring simultaneously two major psychosocial factors, our study is the first step in understanding their independent and combined associations with cognitive health, and may point to new directions for maintaining cognition in the growing population of older African American adults.

## Supplementary Material

igac019_suppl_Supplementary_Material
