# Supplementary material for: Negative and Positive Psychosocial Factors in Relation to Cognitive Health in Older African Americans
Source: Innov Aging. 2022 Apr 1;6(3):igac019. doi: 10.1093/geroni/igac019 (PMC9169895; doi:10.1093/geroni/igac019)
Supplement: igac019_suppl_Supplementary_Material [file igac019_suppl_supplementary_material.docx]

SUPPLEMENTARY MATERIALS

Table S1 page 2

Figure S1 page 3

Table S2 page 4

Table S3 page 5

Table S4 page 6

Table S5 page 7

Supplementary Table S1. Baseline characteristics in the Minority Aging Research Study (MARS) and in African Americans in the Memory and Aging Project (MAP).

| Characteristics | MARS  (n=748) | MAP  (n=109) |
| --- | --- | --- |
| Mean score of purpose in life (SD) | 3.9 (0.5) | 3.7 (0.4) |
| Mean number of depressive symptoms (SD) | 1.3 (1.7) | 1.6 (2.0) |
| Mean age (SD), years | 73.5 (6.3) | 72.4 (7.5) |
| Female, % | 77 | 81 |
| Mean education (SD), years | 14.9 (3.5) | 13.3 (3.1) |
| Antidepressant medication, % | 6 | 15 |
| Mean number of co-morbidities (SD) | 1.6 (1.0) | 1.6 (1.0) |
| Mean number of reported physical activities^a^ (SD) | 1.1 (0.9) | 1.1 (0.9) |
| Smoking status, % |  |  |
| *Never* | 49 | 48 |
| *Former* | 44 | 40 |
| *Current* | 7 | 12 |
| Mean baseline cognitive function (SD), *z*-scores |  |  |
| *Global cognition* | -0.06 (0.52) | -0.18 (0.57) |
| *Episodic memory* | 0.05 (0.58) | 0.03 (0.67) |
| *Semantic memory* | -0.09 (0.76) | -0.17 (0.75) |
| *Visuospatial ability* | -0.34 (0.80) | -0.44 (0.85) |
| *Perceptual speed* | -0.07 (0.76) | -0.31 (0.79) |
| *Working memory* | -0.13 (0.74) | -0.31 (0.81) |

^a^ Number of physical activities (among walking, gardening, and exercise) practiced within the past 2 weeks.

Supplementary Figure 1. Cross-categories of depressive symptoms and purpose in life at baseline and global cognitive function over time^a^


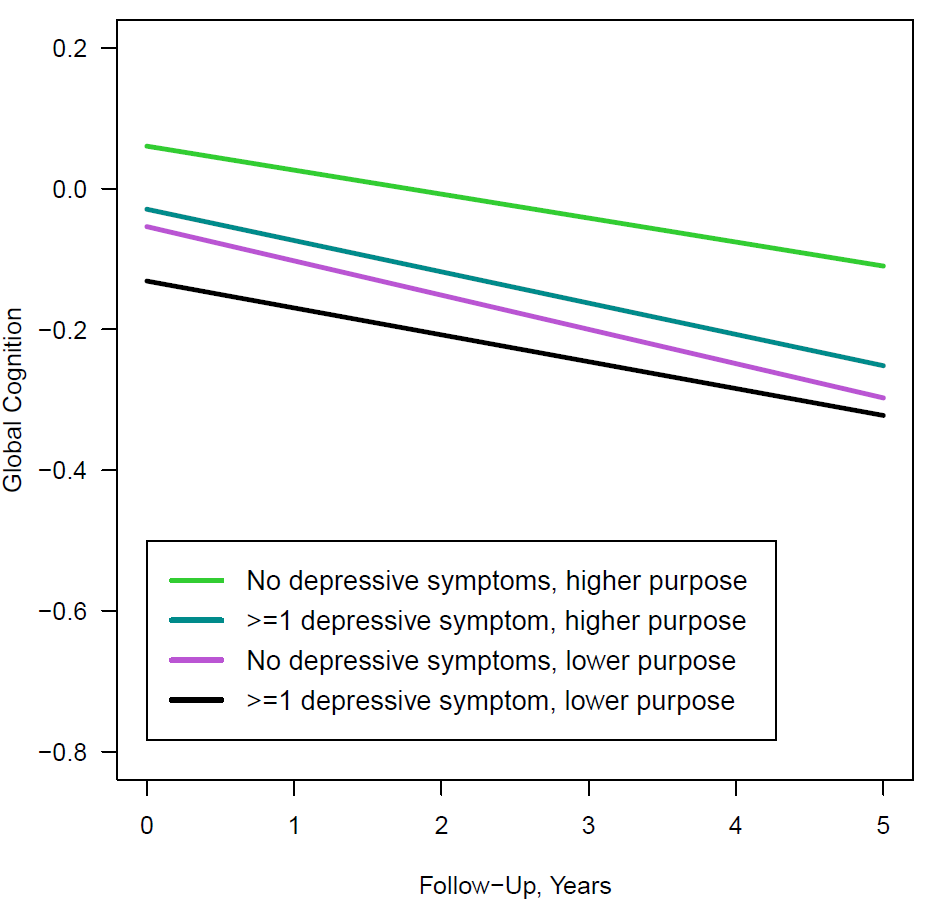


^a^ Trajectories of global cognitive function were plotted for the most common profile of covariates in the study sample (i.e., female in MARS, 73 years of age, 15 years of education, two medical co-morbidities). For greater readability, we did not represent the 95% confidence intervals. Purpose in life was dichotomized using the median score of our analytic sample.

Supplementary Table S2. Multivariable-adjusted mean differences in baseline cognition and cognitive change in five cognitive domains, according to depressive symptoms at baseline.

| Model term | Initial level | | | | | Rate of decline | | |
| --- | --- | --- | --- | --- | --- | --- | --- | --- |
|  | Mean  difference^a^ (SE) | | *P* | Mean  difference^a^ (SE) | | | *P* | |
| Episodic Memory |  |  | | |  | | |  |
| Continuous CES-D score | -0.03 (0.01) | .001 | | | -0.0003 (0.003) | | | .9 |
| 0 symptoms | Ref. | – | | | Ref. | | | – |
| 1 | -0.04 (0.04) | .4 | | | -0.001 (0.01) | | | .9 |
| 2 | -0.11 (0.06) | .07 | | | 0.001 (0.01) | | | .9 |
| ≥ 3 | -0.14 (0.05) | .006 | | | -0.006 (0.01) | | | .6 |
| Semantic Memory |  |  | | |  | | |  |
| Continuous CES-D score | -0.02 (0.01) | .1 | | | -0.004 (0.003) | | | .1 |
| 0 symptoms | Ref. | – | | | Ref. | | | – |
| 1 | -0.11 (0.06) | .04 | | | -0.001 (0.01) | | | .9 |
| 2 | -0.02 (0.08) | .8 | | | -0.004 (0.02) | | | .8 |
| ≥ 3 | -0.15 (0.07) | .02 | | | -0.01 (0.01) | | | .4 |
| Visuospatial ability |  |  | | |  | | |  |
| Continuous CES-D score | -0.01 (0.01) | .5 | | | -0.0002 (0.002) | | | .9 |
| 0 symptoms | Ref. | – | | | Ref. | | | – |
| 1 | 0.03 (0.06) | .6 | | | -0.008 (0.01) | | | .2 |
| 2 | -0.07 (0.08) | .4 | | | -0.002 (0.01) | | | .9 |
| ≥ 3 | -0.04 (0.07) | .5 | | | -0.003 (0.01) | | | .8 |
| Perceptual Speed |  |  | | |  | | |  |
| Continuous CES-D score | -0.03 (0.01) | .046 | | | -0.003 (0.002) | | | .08 |
| 0 symptoms | Ref. | – | | | Ref. | | | – |
| 1 | -0.03 (0.05) | .6 | | | 0.01 (0.01) | | | .5 |
| 2 | -0.09 (0.07) | .2 | | | -0.01 (0.01) | | | .2 |
| ≥ 3 | -0.12 (0.06) | .05 | | | -0.01 (0.01) | | | .4 |
| Working Memory |  |  | | |  | | |  |
| Continuous CES-D score | -0.02 (0.01) | .1 | | | -0.0001 (0.002) | | | .9 |
| 0 symptoms | Ref. | – | | | Ref. | | | – |
| 1 | -0.07 (0.06) | .2 | | | -0.007 (0.01) | | | .3 |
| 2 | -0.13 (0.08) | .09 | | | 0.006 (0.01) | | | .6 |
| ≥ 3 | -0.08 (0.07) | .2 | | | -0.001 (0.01) | | | .9 |

Abbreviations: SE, standard error; CES-D, Center for Epidemiologic Studies Depression scale.

^a^ Adjusted for sex, age at baseline (continuous, in years), education (continuous, in years), cohort, the number of medical co-morbidities, and purpose in life at baseline.

Supplementary Table S3. Multivariable-adjusted mean differences in baseline cognition and cognitive change in five cognitive domains, according to purpose in life at baseline.

| Model term | Initial level | | | | | Rate of decline | | |
| --- | --- | --- | --- | --- | --- | --- | --- | --- |
|  | Mean  difference^a^ (SE) | | *P* | Mean  difference^a^ (SE) | | | | *P* |
| Episodic Memory^b^ |  |  | | |  | |  | |
| Continuous Purpose Score | 0.11 (0.04) | .01 | | | 0.001 (0.01) | | .9 | |
| Quartile 1 | Ref. | – | | | Ref. | | – | |
| Quartile 2 | 0.03 (0.05) | .5 | | | 0.004 (0.01) | | .7 | |
| Quartile 3 | 0.11 (0.05) | .04 | | | -0.01 (0.01) | | .5 | |
| Quartile 4 | 0.16 (0.06) | .004 | | | 0.002 (0.01) | | .9 | |
| Semantic Memory^b^ |  |  | | |  | |  | |
| Continuous Purpose Score | 0.10 (0.06) | .09 | | | -0.01 (0.01) | | .6 | |
| Quartile 1 | Ref. | – | | | Ref. | | – | |
| Quartile 2 | 0.05 (0.06) | .4 | | | -0.01 (0.01) | | .7 | |
| Quartile 3 | 0.12 (0.07) | .07 | | | 0.001 (0.01) | | .9 | |
| Quartile 4 | 0.11 (0.07) | .1 | | | -0.003 (0.01) | | .9 | |
| Visuospatial ability^b^ |  |  | | |  | |  | |
| Continuous Purpose Score | 0.13 (0.06) | .02 | | | -0.005 (0.01) | | .5 | |
| Quartile 1 | Ref. | – | | | Ref. | | – | |
| Quartile 2 | 0.12 (0.06) | .05 | | | 0.001 (0.01) | | .9 | |
| Quartile 3 | 0.17 (0.07) | .01 | | | -0.01 (0.01) | | .2 | |
| Quartile 4 | 0.12 (0.07) | .1 | | | 0.001 (0.01) | | .9 | |
| Perceptual Speed^b^ |  |  | | |  | |  | |
| Continuous Purpose Score | 0.13 (0.05) | .02 | | | -0.001 (0.01) | | .8 | |
| Quartile 1 | Ref. | – | | | Ref. | | – | |
| Quartile 2 | 0.04 (0.06) | .5 | | | -0.004 (0.01) | | .6 | |
| Quartile 3 | 0.15 (0.06) | .02 | | | -0.002 (0.01) | | .8 | |
| Quartile 4 | 0.14 (0.07) | .03 | | | 0.003 (0.01) | | .7 | |
| Working Memory^b^ |  |  | | |  | |  | |
| Continuous Purpose Score | 0.14 (0.06) | .02 | | | 0.01 (0.01) | | .2 | |
| Quartile 1 | Ref. | – | | | Ref. | | – | |
| Quartile 2 | 0.02 (0.06) | .7 | | | -0.001 (0.01) | | .9 | |
| Quartile 3 | 0.03 (0.07) | .7 | | | 0.01 (0.01) | | .3 | |
| Quartile 4 | 0.15 (0.07) | .04 | | | 0.01 (0.01) | | .4 | |

Abbreviations: SE, standard error.

^a^ Adjusted for sex, age at baseline (continuous, in years), education (continuous, in years), cohort, the number of medical co-morbidities, and depressive symptoms at baseline.

^b^ Quartile 1 is 2.0-3.6 points; quartile 2 is 3.7-3.9 points; quartile 3 is 4.0-4.1 points; quartile 4 is 4.2-5.0 points.

Supplementary Table S4. Multivariable-adjusted mean differences in baseline global cognition and global cognitive change according to depressive symptoms and purpose in life at baseline in MARS only (N=748).

| Model term | *n* (%) | Initial level | | | | | Rate of decline | | |
| --- | --- | --- | --- | --- | --- | --- | --- | --- | --- |
|  |  | Mean  difference (SE) | | *P* | Mean  difference (SE) | | | | *P* |
| Depressive symptoms^a,b^ |  |  |  | | |  | |  | |
| Continuous | 748 | -0.02 (0.01) | .04 | | | -0.001 (0.002) | | .7 | |
| 0 | 311 (41.6) | Ref. | – | | | Ref. | | – | |
| 1 | 214 (28.6) | -0.05 (0.04) | .2 | | | 0.006 (0.01) | | .5 | |
| 2 | 80 (10.7) | -0.05 (0.05) | .3 | | | 0.008 (0.01) | | .5 | |
| ≥3 | 143 (19.1) | -0.10 (0.05) | .04 | | | -0.003 (0.01) | | .8 | |
| Purpose in life^a,c^ |  |  |  | | |  | |  | |
| Continuous | 748 | 0.12 (0.04) | .003 | | | -0.0004 (0.01) | | .9 | |
| Quartile 1 [2.0 – 3.6] | 203 (27.1) | Ref. | – | | | Ref. | | – | |
| Quartile 2 [3.7 – 3.9] | 214 (28.6) | 0.01 (0.04) | .9 | | | -0.0001 (0.01) | | .9 | |
| Quartile 3 [4.0 – 4.1] | 161 (21.5) | 0.09 (0.05) | .04 | | | -0.004 (0.01) | | .6 | |
| Quartile 4 [4.2 – 5.0] | 170 (22.7) | 0.14 (0.05) | .003 | | | 0.001 (0.01) | | .9 | |
| Combination^a,d^ | 748 |  | .0002 | | |  | | .7 | |
| ≥1 depressive symptom, lower purpose | 240 (28.0) | Ref. | – | | | Ref. | | – | |
| ≥1 depressive symptom, higher purpose | 197 (23.0) | 0.07 (0.04) | .1 | | | -0.01 (0.01) | | .2 | |
| No depressive symptoms, lower purpose | 112 (13.1) | 0.04 (0.05) | .4 | | | -0.02 (0.01) | | .07 | |
| No depressive symptoms, higher purpose | 199 (23.2) | 0.16 (0.04) | .0001 | | | -0.003 (0.01) | | .7 | |

Abbreviations: SE, standard error.

^a^ Adjusted for sex, age at baseline (continuous, in years), education (continuous, in years), cohort, and the number of medical co-morbidities at baseline.

^b^ Additionally adjusted for the score of purpose in life at baseline.

^c^ Additionally adjusted for the number depressive symptoms at baseline.

^d^ Lower purpose score was dichotomized at the median score obtained in MARS.

Supplementary Table S5. Multivariable-adjusted mean differences in baseline global cognition and global cognitive change according to purpose in life at baseline, among those with no depressive symptoms at baseline.

| Model term | *n* | Initial level | | | | | Rate of decline | | |
| --- | --- | --- | --- | --- | --- | --- | --- | --- | --- |
|  |  | Mean  difference^a^ (SE) | | *P* | Mean  difference^a^ (SE) | | | | *P* |
| Purpose, continuous | 356 | 0.23 (0.06) | <.0001 | | | 0.02 (0.01) | | .2 | |

Abbreviations: SE, standard error.

^a^ Adjusted for sex, age at baseline (continuous, in years), education (continuous, in years), cohort, and the number of medical co-morbidities at baseline.
